# Supplementary material for: Hydrodynamics of transient cell-cell contact: The role of membrane permeability and active protrusion length
Source: PLoS Comput Biol. 2019 Apr 25;15(4):e1006352. doi: 10.1371/journal.pcbi.1006352 (PMC6504115; doi:10.1371/journal.pcbi.1006352)
Supplement: S1 Appendix — Description of computational fluid dynamics method and implementation. Numerical validation of thermal fluctuations and permeability. Description of weighted ensemble method and implementation. (PDF) [file pcbi.1006352.s001.pdf]

Supporting Information S1 Appendix

1 Fluid dynamics simulation

1.1 Model and discretization

As discussed in Methods, the Stochastic Immersed Boundary method formulates the fluid in Eulerian coordinates, which we discretize in a regular Cartesian grid, and the membranes in Eulerian coordinates, which we discretize into a triangular mesh. We describe the membranes using the Helfrich energy functional (1),

$$\Phi_{bend}(\mathbf{X}) = \frac{\kappa_B}{2}\int_{\mathcal{S}} H^2 dA, \tag{1}$$

where κ_B is bending rigidity, H is mean curvature of the membrane surface, and $\mathcal{S}$ is the membrane domain, parameterized by s.

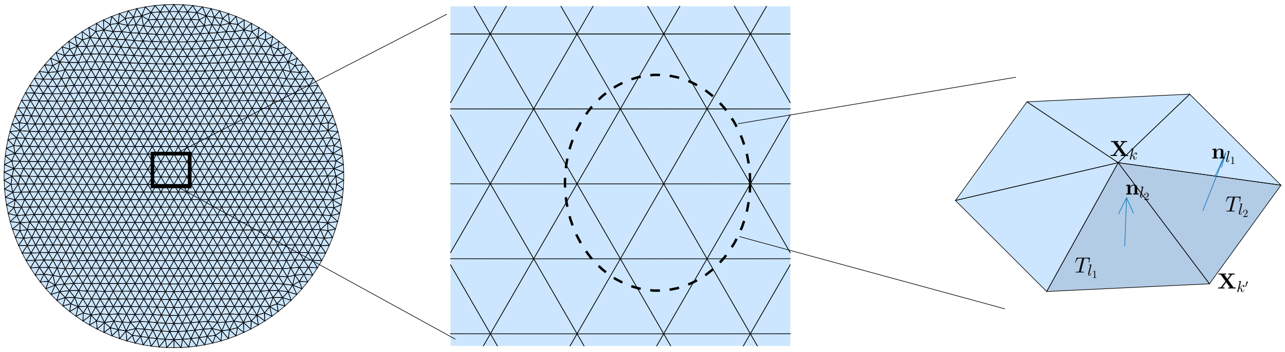

Figure 1: Illustration of vector labeling conventions. Given a vertex $\mathbf{X}_k$ and neighbor vertex $\mathbf{X}_{k'}$, the triangle T_{l_1} and T_{l_2} are in the set of indices of triangles $\tau(k)$, $\mathbf{n}_{l_1}$ and $\mathbf{n}_{l_2}$ are the corresponding normal vectors of triangle T_{l_1} and T_{l_2}.

In the discrete scheme, the curvature is

$$\mathbf{H}^{(k)} = -\sum_{l\in\tau(k)} \frac{1}{2}(\mathbf{n}_l \times \mathbf{E}_l^k). \tag{2}$$

Here k parameterizes the vertex of the triangular mesh, $\tau(k)$ is the set of indices of triangles that touch vertex $\mathbf{X}_k$. The vector E_l^k denotes the edge vector in triangle l opposite vertex k, such that E_l^k points counterclockwise around triangle T_l when viewed from the top, as shown in Fig. 1. The area associated with vertex k is then

$$A^{(k)} = \frac{1}{3}\sum_{l\in\tau(k)} \text{Area}(T_l). \tag{3}$$

The total discrete bending energy is given by

$$\Phi_{bend} = \frac{\kappa_b}{2}\sum_{k=1}^{N_v} \frac{\left\|\mathbf{H}^{(k)}\right\|^2}{A^{(k)}}, \tag{4}$$

where N_v is the number of vertex on the mesh. The force on each node arising from bending resistance is then

$$\mathbf{F}(\mathbf{X}_k) = -\frac{\partial\Phi_{bend}}{\partial\mathbf{X}_k} \tag{5}$$

$$= \frac{\kappa_b}{2}\sum_{l\in\tau(k)}\left(\left(\bar{H}_l - \mathbf{n}_l\cdot\mathbf{C}_l\right)\left(\frac{1}{2}\mathbf{n}_l\times\mathbf{E}_l^k\right) + \frac{1}{2}\mathbf{C}_l\times\mathbf{E}_l^k + \mathbf{n}_l\times\mathbf{h}_l^k\right), \tag{6}$$

where

$$\bar{H}_l = \frac{1}{3}\sum\nolimits_{p\in V(l)} \frac{\left\|\mathbf{H}^{(p)}\right\|^2}{\left(A^{(p)}\right)^2}, \tag{7}$$

$$\mathbf{C}_l = \frac{1}{A_l}\sum\nolimits_{p\in V(l)} \mathbf{E}_p \times \frac{\mathbf{H}^{(p)}}{A^{(p)}}, \tag{8}$$

$$\mathbf{h}_l^k = \frac{\mathbf{H}_k^{(l,3)}}{A_k^{(l,3)}} - \frac{\mathbf{H}_k^{(l,2)}}{A_k^{(l,2)}}. \tag{9}$$

$$\tag{10}$$

In these equations, $V(l)$ is the set of triangles include vertex k, and the notation $()_k^{(l,3)}$ and $()_k^{(l,2)}$ denotes the two vertexes other than k in triangle l, in clockwise order.

In addition to the bending energy, the membrane resists area changes by a surface energy

$$\Phi_{tension}[\mathbf{X}] = \sigma_0 \int_{\mathcal{S}} \left(\frac{dA - dA_0}{dA_0}\right)^2 dA_0 \tag{11}$$

where σ_0 is the surface tension constant. In the discrete scheme, the surface energy is

$$\Phi_{tension} = \sigma_0 \sum_l \left(\frac{A_l(t) - A_l(0)}{A_l(0)}\right)^2 A_0(t), \tag{12}$$

and the resulting force on each node arising from surface tension is

$$\mathbf{F}_{tension}(\mathbf{X}_k) = -\frac{\partial \Phi_{tension}}{\partial \mathbf{X}_k}, \tag{13}$$

$$= \frac{\sigma_0}{2} \sum_{l\in\tau(k)} \left(\frac{A_l(t) - A_l(0)}{A_l(0)}\right) \mathbf{n}_l \times \mathbf{E}_l^k. \tag{14}$$

Finally, the membrane resists shear by a two-dimensional neo-Hookean energy. This is done in previous work using the Immersed Boundary Method to model membranes (2), and is done here not out of physical realism but to maintain stability of the numerical mesh. Let $\mathbf{Z}(\mathbf{q}, t) \in \mathcal{R}^3$ be the reference configuration, and $\mathbf{X}(\mathbf{q}, t)$ the current deformed configuration. the two-dimensional neo-Hookean shear potential is

$$\Phi_{shear}[\mathbf{X}] = \frac{\kappa_s}{2} \int_{\mathcal{S}} \left(\mathrm{trace}(G G_0^{-1}) \left(\frac{\det G}{\det G_0}\right)^{-1/2} - 2\right) (\det G_0)^{1/2}\, d\mathbf{q}, \tag{15}$$

where κ_s is the shear modulus of the membrane. Here the Cauchy-Green deformation tensors are defined as

$$G = \left(\frac{\partial \mathbf{X}}{\partial \mathbf{q}}\right)^T \left(\frac{\partial \mathbf{X}}{\partial \mathbf{q}}\right), \quad G_0 = \left(\frac{\partial \mathbf{Z}}{\partial \mathbf{q}}\right)^T \left(\frac{\partial \mathbf{Z}}{\partial \mathbf{q}}\right). \tag{16}$$

The discrete shear energy of triangle l reads as

$$\Phi_{shear}(T_l) = \kappa_s \left(\frac{\|\mathbf{X}_{13}\|^2 \|\mathbf{Z}_{23}\|^2 - 2(\mathbf{X}_{13}\cdot\mathbf{X}_{23})(\mathbf{Z}_{13}\cdot\mathbf{Z}_{23}) + \|\mathbf{X}_{23}\|^2 \|\mathbf{Z}_{13}\|^2}{8Area(T_{\mathbf{X},l})}\right) - \kappa_s Area(T_{\mathbf{Z},l}). \tag{17}$$

So the force can be found as

$$\mathbf{F}_1 = -\kappa_s \frac{\|\mathbf{Z}_{23}\|^2 \mathbf{X}_{13} - 2(\mathbf{Z}_{23}\cdot\mathbf{Z}_{13})\mathbf{X}_{23}}{4Area(T_{\mathbf{X},l})} - \kappa_s' \frac{\|\mathbf{X}_{23}\|^2 \mathbf{X}_{13} - 2(\mathbf{X}_{23}\cdot\mathbf{X}_{13})\mathbf{X}_{23}}{4Area(T_{\mathbf{X},l})} \tag{18}$$

$$\mathbf{F}_2 = -\kappa_s \frac{\|\mathbf{Z}_{23}\|^2 \mathbf{X}_{33} - 2(\mathbf{Z}_{23}\cdot\mathbf{Z}_{13})\mathbf{X}_{23}}{4Area(T_{\mathbf{X},l})} - \kappa_s' \frac{\|\mathbf{X}_{23}\|^2 \mathbf{X}_{33} - 2(\mathbf{X}_{23}\cdot\mathbf{X}_{13})\mathbf{X}_{23}}{4Area(T_{\mathbf{X},l})} \tag{19}$$

$$\mathbf{F}_3 = -\kappa_s \frac{\|\mathbf{Z}_{22}\|^2 \mathbf{X}_{31} - 2(\mathbf{Z}_{21}\cdot\mathbf{Z}_{31})\mathbf{X}_{21}}{4Area(T_{\mathbf{X},l})} - \kappa_s' \frac{\|\mathbf{X}_{21}\|^2 \mathbf{X}_{31} - 2(\mathbf{X}_{21}\cdot\mathbf{X}_{31})\mathbf{X}_{21}}{4Area(T_{\mathbf{X},l})} \tag{20}$$

where

$$\kappa_s' = -\frac{\kappa_s}{8}\left(\frac{\|\mathbf{X}_{13}\|^2\|\mathbf{Z}_{23}\|^2 - 2(\mathbf{X}_{13}\cdot\mathbf{X}_{23})(\mathbf{Z}_{13}\cdot\mathbf{Z}_{23}) + \|\mathbf{X}_{23}\|^2\|\mathbf{Z}_{13}\|^2}{Area(T_{\mathbf{X},l})^2}\right) \tag{21}$$

Since this shear resistance is artificial, we wish to minimize its impact on the simulation dynamics. To do so, we choose a shear modulus that is small enough to make the shear energy much smaller than bending and stretching energies, specifically $\kappa_s = 0.1\text{N/m}^2$. We also change the reference configuration to the current configuration gradually by endowing $\mathbf{Z}$ with dynamics $\mathbf{Z}_{\mathbf{K}}^{n+1} = (1-c_{ref})\mathbf{X}_{\mathbf{K}}^{n+1} + c_{ref}\mathbf{Z}_{\mathbf{K}}^{n}$, where c_{ref} with $c_{ref} = 0.9$, so that approximately every 10 timesteps, the reference shear configuration has been "reset".

The two membranes are connected by approximately inextensible springs, which therefore exert an equal-and-opposite pair of external forces on each membrane via $\mathbf{F}_{ex}$. These springs are placed on every pair of nodes, except for a disk of radius r_{free} centered on the membrane. Therefore, outside the center free region, their intermolecular spacing is initially Δx. The springs are Hookean with spring constant $k_{\text{adh}} = 5\times 10^2\text{pN}/\mu\text{m}$.

1.2 Numerical implementation

We use Eulerian and Lagrangian reference frames for the fluid and structure, respectively (2, 3). The Eulerian fluid domain $\{\mathbf{x}|\mathbf{x}\in\Omega\}$ is resolved by a finite difference discretization. The fluid variables (velocity field $\mathbf{u}$ and pressure field p) are represented on a periodic grid with length L along each direction, N grid points along each direction, and grid spacing $\Delta x = L/N$. The discrete Fourier transform of the fluid variables are

$$\hat{\mathbf{u}}_{\mathbf{k}} = \frac{1}{N^3}\sum_{\mathbf{m}} \mathbf{u}_{\mathbf{m}} e^{-2\pi i\mathbf{k}\cdot\mathbf{m}/N}. \tag{22}$$

$$\mathbf{u}_{\mathbf{m}} = \sum_{\mathbf{k}} \hat{\mathbf{u}}_{\mathbf{k}} e^{2\pi i\mathbf{k}\cdot\mathbf{m}/N}. \tag{23}$$

Here each sum runs over the N^3 lattice points defined by $\mathbf{m} = \{m_1, m_2, m_3\}$ and $\mathbf{k} = \{k_1, k_2, k_3\}$, where $0 \le m_i \le N-1$ and $0 \le k_i \le N-1$ for $i = 1, 2, 3$. The membranes in the Lagrangian domain $\{\mathbf{X}|\mathbf{X}\in\mathcal{S}\}$ are also resolved by a evenly spaced finite difference discretization.

Solving unsteady Stokes equations in the Fourier space, the velocity is (2, 4)

$$\hat{\mathbf{u}}_{\mathbf{k}}(t^{n+1}) = \hat{\mathbf{u}}_{\mathbf{k}}(t^n)e^{-\alpha_{\mathbf{k}}\Delta t} + \frac{(1-e^{-\alpha_{\mathbf{k}}\Delta t})}{\rho\alpha_{\mathbf{k}}}\xi_{\mathbf{k}}\hat{\mathbf{f}}_{\mathbf{k},total} + \sqrt{2D_{\mathbf{k}}}\xi_{\mathbf{k}}\int_{t^n}^{t^{n+1}} e^{-\alpha_{\mathbf{k}}(t^{n+1}-s)}d\tilde{B}_{\mathbf{k}}(s), \tag{24}$$

where

$$\alpha_{\mathbf{k}} = \frac{2\eta}{\rho\Delta x^2}\sum_{l=1}^{3}\left[1-\cos\left(2\pi\mathbf{k}^{(l)}/N\right)\right], \tag{25}$$

and $\xi_{\mathbf{k}} = I - \hat{\mathbf{g}}_{\mathbf{k}}\cdot\hat{\mathbf{g}}_{\mathbf{k}}^T/|\hat{\mathbf{g}}_{\mathbf{k}}|^2$ is the projection orthogonal to $\hat{\mathbf{g}}_{\mathbf{k}}$, defined by

$$\hat{\mathbf{g}}_{\mathbf{k}} = \sin\left(2\pi\mathbf{k}^{(l)}/N\right)/\Delta x, \tag{26}$$

which is used to enforce the incompressibility constraint. The strength of the thermal fluctuation is described by $D_{\mathbf{k}}$, which is (2, 4)

$$D_{\mathbf{k}} = \begin{cases} \frac{k_BT}{2\rho L^3}\alpha_{\mathbf{k}}, & \text{for } \mathbf{k}\in\mathcal{H}, \\ \frac{k_BT}{\rho L^3}\alpha_{\mathbf{k}}, & \text{for } \mathbf{k}\notin\mathcal{H}. \end{cases} \tag{27}$$

Here $\mathcal{H} = \{\mathbf{k}|k_i = 0, N/2, i = 1, 2, 3\}$. The structure is updated by

$$\mathbf{X}^{n+1,(k)} = \mathbf{X}^{n,(k)} + \sum_{\mathbf{m}}\delta_c(\mathbf{x}_{\mathbf{m}} - \mathbf{X}^{\mathbf{n},k})h^3\mathbf{H}_{\mathbf{k}}^n + \Delta t\psi\left(\mathbf{F}_{mem}^{n,(k)} + \mathbf{F}_{ex}^{n,(k)} + \mathbf{F}_{thm}^{n,(k)}\right) \tag{28}$$

where Δt is the step size, and

$$\mathbf{H}_{\mathbf{k}}^n = \int_{t_n}^{t_{n+1}} \mathbf{u}_{\mathbf{k}}(s)ds, \tag{29}$$

where $t_n = n\Delta t$ and

$$\mathbf{F}_{thm}^{n,(k)} = \sqrt{\frac{2k_BT}{\Delta t\psi}}\mathcal{N}, \tag{30}$$

where $\mathcal{N}$ is a three-dimensional real-valued Gaussian random variable with independent components, each of which has mean 0 and variance 1 (2).

To update the position of the structure, $\hat{\mathbf{H}}_k^n$ can be calculated in the Fourier space by

$$\hat{\mathbf{H}}_{\mathbf{k}}^n = \left(\frac{1-e^{-\alpha_{\mathbf{k}}\Delta t}}{\alpha_k}\right)\hat{\mathbf{u}}_{\mathbf{k}}^n + \left(\Delta t - \frac{1-e^{-\alpha_{\mathbf{k}}\Delta t}}{\alpha_k}\right)\rho^{-1}\xi_{\mathbf{k}}\hat{\mathbf{f}}_{\mathbf{k},total}^n$$
$$-\frac{\sqrt{2D_{\mathbf{k}}}}{\alpha_{\mathbf{k}}\Delta t}\int_{t^n}^{t^{n+1}} e^{-\alpha_{\mathbf{k}}(t^{n+1}-u)}\xi_{\mathbf{k}}d\tilde{B}_{\mathbf{k}}(u) + \frac{\sqrt{2D_{\mathbf{k}}}}{\alpha_{\mathbf{k}}\Delta t}\left(\xi_{\mathbf{k}}\hat{B}_{\mathbf{k}}(t_{n+1}) - \xi_{\mathbf{k}}\hat{B}_{\mathbf{k}}(t_n)\right). \tag{31}$$

And the covariance structure of $\int_{t^n}^{t^{n+1}} e^{-\alpha_{\mathbf{k}}(t-s)}\xi_{\mathbf{k}}d\mathbf{B}_{\mathbf{k}}(s)$ and $\left(\xi_{\mathbf{k}}\mathbf{B}_{\mathbf{k}}(t^{n+1}) - \xi_{\mathbf{k}}\mathbf{B}_{\mathbf{k}}(t^n)\right)$ reads as

$$\mathbb{E}\left[\mathrm{Re}\left(\int_{t^n}^{t^{n+1}} e^{-\alpha_{\mathbf{k}}(t^{n+1}-s)}d\mathbf{B}_{\mathbf{k}}(s)\right)\mathrm{Re}\left(\mathbf{B}_{\mathbf{k}}(t^{n+1}) - \mathbf{B}_{\mathbf{k}}(t^n)\right)\right] = \frac{1}{\alpha_{\mathbf{k}}}(1-e^{-\alpha_{\mathbf{k}}\Delta t}). \tag{32}$$

We refer to (2) for more details about the discretization of the bending energy, surface energy, and the corresponding elastic force.

Note that in the above formulation, the $k = 0$ mode evolves with a dynamic equation similar to the other modes. There is a net external force in simulations with active force (applied to nodes near the center of one of the membranes) which would result in net motion of the fluid. However, a counter-acting force is also applied to the nonspecific adhesions to ensure that the center-of-mass of the membranes do not drift. This counter-acting force on the adhesions is biophysically realistic, since an adherent cell that pushes "down" with a protrusion will increase the tension on its adhesions by an equal amount.

The fluid domain is a rectangular box of $512 \times 512 \times 192$, where the spatial size $\Delta x = 5$ nm. The average of the side length of the triangular mesh is $\Delta L = 10$ nm. We set the time step $\Delta t = 0.5$ ns. We used NVIDIA GPU Tesla K40 and Geforce 1080 Ti. The average machine time cost per step is 0.1 s, and the CUDA C/C++ program is 5-10 times faster than the MATLAB version.

1.3 Numerical validation

1.3.1 Equilibrium distribution

The thermal equilibrium for an undulating sphere has been previously solved for analytically, in the limit of small deformations (5). Therefore, as an initial test of the thermal fluctuations in our code, we simulate a sphere and compare to the quasi-spherical theory.

The spherical harmonic expansion of a nearly spherical vesicle shape is

$$R(\theta,\phi) = R\left(1 + \sum_{l\geq 0}^{l_{max}}\sum_{m=-l}^{l} u_{l,m}Y_{l,m}(\theta,\phi)\right). \tag{33}$$

where R is the equivalent radius, $Y_{l,m}(\theta,\phi)$ is spherical harmonics, and $u_{l,m}$ is the corresponding nondimensional amplitude of deformation, where we assume $|u_{l,m}| \ll 1$. Each mode in the above expansion satisfies a Langevin equation (5),

$$\partial_t u_{l,m} = -\frac{\kappa_b}{\eta R^3}E_l F_l u_{l,m} + \zeta_{l,m}, \tag{34}$$

where

$$E_l = (l+2)(l-1)\left[l(l+1) + \frac{R^2}{\kappa_b}\Sigma_0\right], \quad F_l = \frac{l(l+1)}{4l^3+6l^2-1} \tag{35}$$

and Σ_0 is the isotropic membrane tension. In thermal equilibrium, these modes should satisfy (5),

$$\lim_{t\to\infty}\langle|u_{l,m}|^2\rangle = \frac{k_B T}{\kappa_b E_l}. \tag{36}$$

In our simulations, we use the triangle faces to calculated the amplitude of deformation for each spherical harmonics,

$$u_{l,m}(t_k) = \sum_{i\in T}\sin\theta_i r(\theta_i,\phi_i,t_k)Y_l^m(\theta_i,\phi_i)\cos(\xi_i)dA_i/|\mathbf{x}_{i,c}|^2 \tag{37}$$

where ξ_i is the angle between the center of the i-th triangle $\mathbf{x}_{i,c}$ and its normal direction $\mathbf{n}_i$,

$$\cos(\xi_i) = \frac{\mathbf{x}_{i,c} \cdot \mathbf{n}_i}{|\mathbf{x}_{i,c} \cdot \mathbf{n}_i|}, \tag{38}$$

In Fig. 2, we compare the mode decomposition to the analytical theory from (5). We find good agreement for each mode, with consistent underestimates for $m \neq 0$. We attribute these underestimates to the manner in which we numerically extract the modes from the discrete mesh, which consistently underestimates distances from the sphere's center.

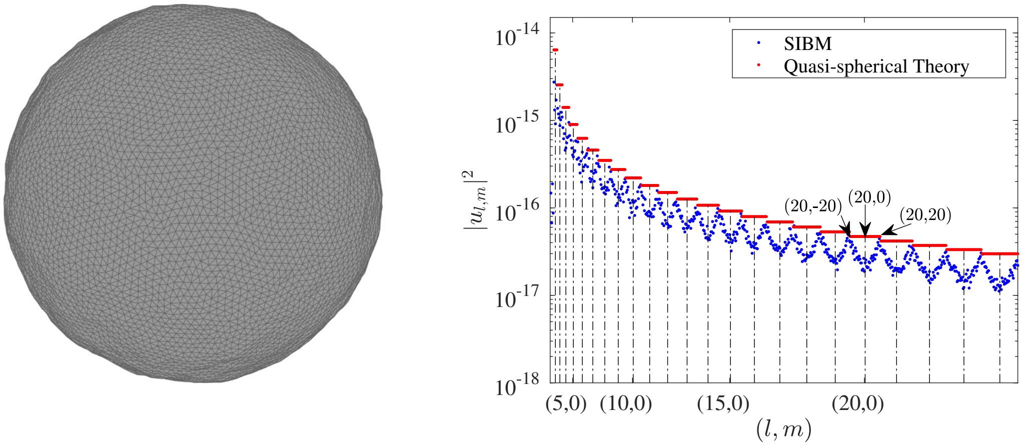

Figure 2: (Left) Simulation of thermally undulating sphere. (Right) Amplitude of each spherical harmonic mode of deformation. Here the values of (l, m) are denoted on the same axis (so the horizontal axis corresponds to $l^2 + l + m + 1$, and the order is $l = 1, 2, 3, ...$ and $m = -l, -l+1, ..., l-1, l$ for each l). In this test, we set $R = 13.7\mu$m, $\kappa_b = 8.7 \times 10^{-20}$ J, and $\kappa_s = 1.2 \times 10^{-4}$ N/m. Note that almost all modes are underestimated because we use the center position of the triangular surface instead of vertex.

1.3.2 Permeability

The membrane permeability included in the equation of motion for the membranes is proportional to a permeability coefficient, ψ, that describes the relationship between pressure and the difference in velocity of the fluid and the structure, with units of nm/s Pa. To validate the numerical implementation of this permeability, we simulate a disk subject to a uniform pressure P, and measure the velocity difference between fluid at the membrane and the membrane's velocity, Δu. We do this for 5 different pressure values and two different permeabilities, shown in Fig. 3.

In agreement with the permeability law we attempt to model, we find a linear relationship between pressure and velocity difference. The slope of this line should be equal to the permeability coefficient. We find that this is approximately true within 30%, a discrepancy we attribute to the fact that objects in the immersed boundary framework have an effective radius larger than their explicit radius (in this case, the explicit radius is 40nm, while the permeability coefficient implies an effective radius of about 50nm).

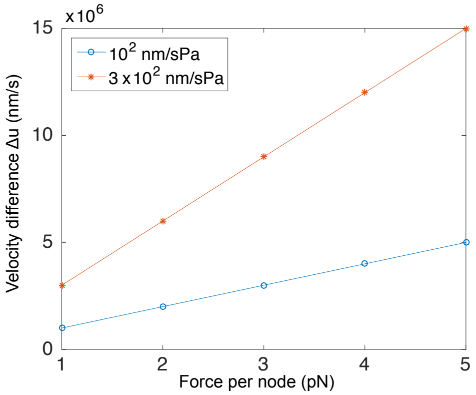

Figure 3: We test the permeability model by pulling a disk applying equal the same force on each node perpendicular to the disk plane. Here $\mathbf{\Delta u} = \mathbf{u} - \mathbf{u}_0$, where $\mathbf{u}_0$ is the average velocity of the membrane and $\mathbf{u}$ is the average fluid velocity at the membrane. The radius of the disk is 40 nm, and there are overall 88 nodes on the plane.

2 Rare event simulation

2.1 Overview of rare event simulation method

The Weighted Ensemble (WE) algorithm (6) is a method for efficient simulation of rare events. The method is broadly applicable to stochastic dynamic models and simulation engines (7). Details of the methodology are discussed in a recent review (8) and references therein. Briefly, the algorithm works as follows: state-space is divided up into bins that span rare transitions of interest, along a progress coordinate (λ). Initially, a single simulation trajectory, or "replica", is assigned a weight of 1 and allowed to freely move within and between bins for a user-defined lagtime τ_{WE}. After each iteration of τ_{WE}, a splitting and culling procedure divides and/or combines replicas and their associated weights in such a way as to reach and maintain an approximately equal number of weighted replicas, M, in each bin. Over the course of the simulation, the combined weights of the replicas in a bin (averaged over successive iterations) will evolve toward the steady-state probability of the system to reside in that bin. The mean first passage time from an initial region of interest A to a target region B is obtained as

$$\mathrm{MFPT}_{A\to B} = \frac{1}{\overline{\Phi}_{A\to B}}, \tag{39}$$

where $\overline{\Phi}_{A\to B}$ is the is the steady-state, time-averaged probability flux into region B of trajectories that were previously in A. This flux is estimated from the weights of replicas that successfully reach the target B; upon reaching the target, these weights are then immediately reintroduced into region A for the next simulation iteration.

By maintaining even sampling in each bin, with weights proportional to probability, the algorithm essentially redistributes computational effort from high- to low-probability regions. The method furthermore increases efficiency by capitalizing on incremental progress of replicas toward the target in many, short-time simulations. By contrast, conventional simulation requires long waiting-times for individual trajectories to fully complete a rare transition of interest.

2.2 Simulation of rare events in Ornstein-Uhlenbeck process

Although several variants of the WE method exists (8), we use the original implementation by Huber and Kim (6), which is often termed the out-of-equilibrium method because replicas which enter B are continuously reintroduced into A.

To model the full fluid dynamics simulations presented in the Main Text, we use a one-component Ornstein-Uhlenbeck (OU) process to describe a single membrane,

$$dZ = \frac{1}{\tau} Z dt + \frac{\sigma}{\sqrt{\tau}} dW, \tag{40}$$

and a two-component OU process to describe the interface,

$$dX = \frac{1}{\tau_{\mathrm{slow}}} X dt + \frac{\sigma}{\sqrt{\tau_{\mathrm{slow}}}} dW_1, \tag{41}$$

$$dY = \frac{1}{\tau_{\mathrm{fast}}} Y dt + \frac{\sigma}{\sqrt{\tau_{\mathrm{fast}}}} dW_2, \tag{42}$$

$$Z = cX + \sqrt{1-c^2} Y. \tag{43}$$

For the one-component OU process, a one-dimensional binning over Z is used to subdivide state space and track progress of the trajectories. For the two-component OU process, a 2D binning over X and Y is used. The target region B is defined as $Z(t) \geq Z^*$ and A is any $Z(t) < Z^*$. The binning strategy, number of replicas per bin (M) and iteration time (τ_{WE}) were chosen through trial simulations in such a way as to maximize simulation efficiency while minimizing iteration-to-iteration fluctuations of the estimated flux, $\Phi_{A\to B}$. The WE code was implemented in MATLAB. Simulation parameters are given in Table 1.

2.3 Weighted Ensemble results

We applied the Weighted Ensemble algorithm to the one-component and two-component OU processes with parameters in Table 1. The one-component calculations show good agreement (Main Text Fig. 4) to an analytical

Table 1: Ornstein-Ulhenbeck approximation parameters and Weighted Ensemble computational parameters used to approximate mean first-passage times

	Model Parameters	Simulation Parameters
One-component OU	$\sigma = 3.1248$ nm,	$dt = 5.0 \times 10^{-10}$
	$\tau_{OU} = 1.049 \times 10^{-6}$	
	$Z^* = \{9, 15, 20, 25, 30\}$	$\tau_{WE} = 5.0 \times 10^{-9}$,$M = 50$,Bins= $[-\infty, -15, -10, -5, 0, 3 : 0.2 : Z^*]$
Two-component OU	$\sigma = 4.5713$ nm	$dt = 2.5 \times 10^{-10}$
	$\tau_{\text{slow}} = 8.183 \times 10^{-5}$ nm,	
	$\tau_{\text{fast}} = 5.215 \times 10^{-7}$ nm	
	$Z^* = \{9, 15, 20, 25\}$	$\tau_{WE} = 1.0 \times 10^{-7}$, $M = 50$, Bins$_x$ =Bins$_y$ = $[-\infty, -10 : 2 : Z^*]$

approximation (9) for the MFPT of a simple OU process starting from $Z_0 = 0$ to reach $Z(t) \geq Z^*$, specifically

$$\text{MFPT}(Z^*) = \tau\sqrt{2\pi}\int_0^{Z^*/\sigma} e^{u^2/2}du. \tag{44}$$

As a validation, we simulate the two-component process at various values of c. The fraction of the process driven

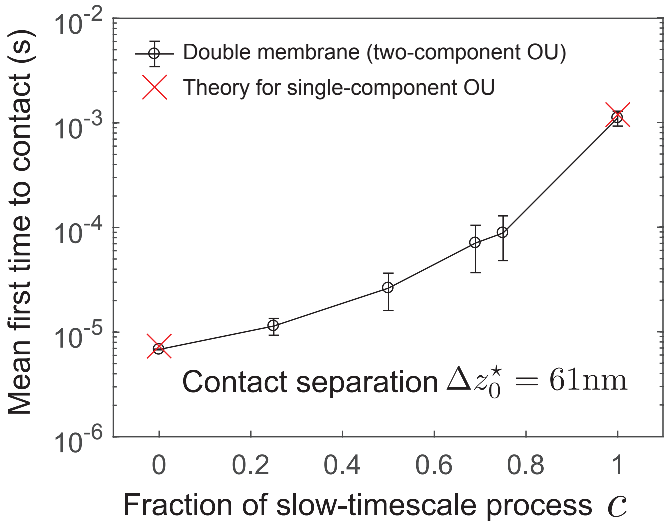

Figure 4: Validation of MFPTs for two-component OU at $Z^\star = 9$nm (corresponding to $\Delta z_0^\star = 61$nm. Red crosses indicate analytical results from (9).

by the slow process, c (defined in Eq. 43), dictates the MFPT. In addition, since $c = 0$ and $c = 1$ are effectively single-component OU processes, we can validate our computation with the theoretical MFPTs from (9).

References

[1] Helfrich W. Elastic properties of lipid bilayers: theory and possible experiments. Z Naturf C. 1973;28:693–703.

[2] Wu CH, Fai TG, Atzberger PJ, Peskin CS. Simulation of osmotic swelling by the stochastic immersed boundary method. SIAM J Sci Comp. 2015;37(4):B660–B688.

[3] Atzberger P. Stochastic Eulerian Lagrangian Methods for Fluid Structure Interactions with Thermal Fluctuations. J Comp Phys. 2011;230:2821–2837.

[4] Atzberger PJ. A note on the correspondence of an immersed boundary method incorporating thermal fluctuations with Stokesian-Brownian dynamics. Physica D. 2007;226:144–150.

[5] Seifert. Fluid membranes in hydrodynamic flow fields: Formalism and an application to fluctuating quasispherical vesicles in shear flow. Eur Phys J B. 1999;8:405-415

[6] Huber GA, Kim S. Weighted-ensemble Brownian dynamics simulations for protein association reactions. Biophys J. 1996;70(1):97–110.

[7] Zhang BW, Jasnow D, Zuckerman DM. The weighted ensemble path sampling method is statistically exact for a broad class of stochastic processes and binning procedures. J Chem Phys. 2010;132(5):054107.

[8] Zuckerman DM, Chong LT. Weighted Ensemble Simulation: Review of Methodology, Applications, and Software. Annu Rev Biophysics. 2017;46(1):43–57.

[9] Thomas MU. Some mean first-passage time approximations for the Ornstein-Uhlenbeck process. J Applied Probability. 1975;12(3):600–604.

[10] Atzberger PJ Incorporating Shear into Stochastic Eulerian Lagrangian Methods for Rheological Studies of Complex Fluids and Soft Materials. Physica D. 2013; 265:57–70.
